# Do glial‐reactivity and cerebral blood flow modulate cerebral glucose metabolism in Alzheimer’s disease?

**DOI:** 10.1002/alz.088897

**Published:** 2025-01-09

**Authors:** Joseph Nowell, Grazia Daniela Femminella, Craig Ritchie, Clive Holmes, Zuzana Walker, Basil H Ridha, Robert M Lawrence, Brady McFarlane, Hilary Archer, Elizabeth Coulthard, Benjamin R Underwood, Paul Koranteng, Salman Karim, Aparna Prasanna, Kehinde Junaid, Bernadette McGuinness, Ramin Nilforooshan, Simon Thacker, Gregor Russell, Naghma Malik, Vandana Mate, Lucy Knight, Sajeev Kshemendran, Christian Holscher, John E Harrison, Rainer Hinz, George Tadros, Anthony Peter Passmore, Clive G Ballard, Paul Edison

**Affiliations:** ^1^ Imperial College London, London UK; ^2^ Edinburgh Dementia Prevention, University of Edinburgh, Edinburgh UK; ^3^ University of Southampton, Southampton UK; ^4^ University College London, London UK; ^5^ Dementia Research Centre, Institute of Neurology, University College London, London UK; ^6^ South West London and St George's Mental Health NHS Trust, London UK; ^7^ Southern Health NHS Foundation Trust, Southampton UK; ^8^ North Bristol NHS Trust, Bristol UK; ^9^ University of Bristol, Bristol UK; ^10^ University of Cambridge, Cambridge, Cambridgeshire UK; ^11^ Northamptonshire Healthcare NHS Foundation Trust, Northampton UK; ^12^ Lancashire Care NHS Foundation Trust, Preston UK; ^13^ Black Country Partnership NHS Foundation Trust, Wolverhampton UK; ^14^ Nottinghamshire Healthcare NHS Foundation Trust, Nottingham UK; ^15^ Centre for Public Health, Queen's University Belfast, Belfast, County Antrim UK; ^16^ Surrey and Borders Partnership NHS Foundation Trust, Chertsey UK; ^17^ Derbyshire Healthcare NHS Foundation Trust, Derby UK; ^18^ Bradford District Care NHS Foundation Trust, Bradford UK; ^19^ North West Boroughs Partnership NHS Foundation Trust, Warrington UK; ^20^ Cornwall Partnership NHS Foundation Trust, Redruth UK; ^21^ Somerset Partnership NHS Foundation Trust, South Petherton UK; ^22^ South Staffordshire and Shropshire Healthcare NHS Foundation Trust, Shrewsbury UK; ^23^ Lancaster University, Lancaster UK; ^24^ Alzheimer Center Amsterdam, Amsterdam UMC, Vrije Universiteit Amsterdam, Amsterdam Netherlands; ^25^ University of Manchester, Manchester UK; ^26^ Heart of England NHS Foundation Trust, Birmingham UK; ^27^ Queen's University Belfast, Belfast UK; ^28^ University of Exeter, Exeter, Devon UK

## Abstract

**Background:**

Alzheimer’s disease is a devastating neurodegenerative disorder with a complex pathogenesis. One main pathological feature utilised in diagnosis is neurodegeneration or neuronal injury, which is reflected in reductions in cerebral glucose metabolism measured by [18F]Fluorodeoxyglucose ([18F]FDG) positron emission tomography (PET). Here we evaluated the involvement of glial reactivity measured with magnetic resonance spectroscopy (MRS) and cerebral blood flow measured with arterial spin labelling (ASL) on [18F]FDG PET as a measure of cerebral glucose metabolism.

**Method:**

123 people living with early Alzheimer’s disease who completed baseline evaluations on the evaluating liraglutide in Alzheimer’s disease trial were enrolled. Participants completed [18F]FDG PET scans with arterial input, T1 weighted MRI, single‐voxel ^1^HMRS, and pulsed ASL scans at Imperial College London Clinical Imaging Facility. The Totally Automatic Robust Quantitation in NMR (TARQUIN) package was used to process MRS scans and identify the concentration of myo‐inositol within the posterior cingulate cortex (PCC), a marker of glial activation. Oxford‐ASL was utilised to process ASL and quantify cerebral blood flow in the PCC. Finally, spectral analysis was performed on the [18F]FDG PET scans to assess the cerebral metabolic rate of glucose in the PCC.

**Result:**

Pearson’s correlations were performed between the cerebral metabolic rate of glucose, cerebral blood flow and glial activity measured by the level of myo‐inositol in the PCC. Increased cerebral glucose metabolism was correlated with higher myo‐inositol in this sample of Alzheimer’s disease participants. In contrast, cerebral blood flow was not associated with cerebral glucose metabolism.

**Conclusion:**

Here we demonstrate that increased glial reactivity contributes to [18F]FDG PET signal in the early stages of Alzheimer’s disease. In response to early neuronal injury, astrocytes and microglia may become activated and enhance regional rates of glucose consumption. Hence, the contribution from these cells in addition to neurons should be considered in interpreting [18F]FDG PET as a measure of cerebral glucose metabolism. Interestingly, cerebral blood flow did not influence glucose metabolism. Microglia and astrocyte reactivity may contribute to an increase the cerebral glucose metabolism while neuronal loss and synaptic function may contribute to lower glucose metabolism measured by [18F]FDG in the early stages of Alzheimer's disease.

